# Bulletin of the Buffalo General Hospital

**Published:** 1912-04

**Authors:** 


**Bulletin of the Buffalo General Hospital**

VOL. I. APRIL, 1912. No. IV.

### Resolutions

AT a special meeting of the Board of Trustees of the Buffalo General Hospital, held immediately after the death of Mr. Pardee, the following memorial was adopted:

BY the death of Charles W. Pardee, The Buffalo General Hospital has been deprived of the services of one of the most valued and efficient officers with which it has been blessed in the half century of its work. During that time many men of marked ability have given largely of their valuable time and services to the work of the Hospital, and have served with fidelity and zeal, but among them all, the signal services and extraordinary and continued labors of Mr. Pardee stand out prominently. Mr. Pardee acted as Secretary and Trustee during the years 1888 to 1891, and in the year 1904 was again elected Trustee and President of the institution, serving continuously in that capacity up to the day of his death.

During all that time his interest in and ardor for the institution knew no bounds and suffered no lapse. He gave unsparingly at all times of his means, and his personal attention and services were constantly and continuously applied to every interest and detail of the institution.

During his service and under his personal direction the hospital made remarkable progress in all lines of its work and equipment. Many improvements owe their existence to his initiative. He was at all times a close student of all matters pertaining to the betterment and conduct of hospitals, and applied himself to the scientific and technical work in connection therewith. Prominent among his ambitions for the hospital was the beautifying of the buildings and grounds, and much was accomplished along these lines under his direction. These, together with the pathological laboratory installed at his expense, will continue to stand out prominently among his many benefactions.

His work continued unabated up to the day of his death, and this Board desires to express its deep sense of personal loss, as well as the great loss suffered by the institution.

Resolved, that this memorial be spread upon the minutes of this meeting, and an engrossed copy sent to his family, together with the sympathy of this Board.

The following resolutions were adopted by the staff:

AT a meeting of the Staff of The Buffalo General Hospital, March 18, 1912, the following memorial was formally adopted:

The Medical Staff of The Buffalo General Hospital has realized ever since Mr. Charles W. Pardee became President of its Board of Trustees, how greatly they were indebted to him, not only for the cordial and reciprocally helpful support which he has ever accorded to them, but for a deeper and more sympathetic appreciation of our efforts toward our common purpose than is usually expressed by lay officers of a medical organization or hospital.

We feel that, in so far as was possible, he has realized our needs and ambitions, and know quite well, that his purse has ever been open, when hospital funds were insufficient or unavailable.

We realize, too, that it is largely due to his untiring energy and ceaseless zeal, that the Hospital has been able to accomplish so much, and to be entitled to its present position among similar institutions of this country: a position in which we all take the greatest pride. Few officers have been willing to give daily and constantly of their time as did Mr. Pardee, to the minutest details.

We understand something of the efforts which have been required on his part to effect many of our recent improvements, and to place the institution upon a satisfactory financial footing.

From every direction then, we are made to feel, what a serious blow has fallen upon us and upon our Hospital, and feel that we should be unjust to ourselves and to Mr. Pardee's memory, did we not spread upon our minutes this testimonial to the great value alike to the institution and to the community at large of those services which he so capably and so generously rendered.

And we wish, not only to transmit to Mrs. Pardee and his bereaved family our expressions of deepest sympathy, but to give public utterance to our own feelings and of our own appreciation of his devotion to the Hospital.

---

### In the Pardee Laboratory.

UPON entering the hospital, each case has an analysis made of a single sample of urine. This analysis is made and the report written on the chart the next morning, so that the attending physician or surgeon is informed when making rounds.

The morning after the patient's arrival at the hospital a twenty-four hour sample is started. In its analysis, the total amount is measured, color noted, reaction taken, specific gravity, total solids and urea estimated; tests for sugar and albumen are made and the per cent. given; the presence of indican and, at times, acetone, and the amount of chlorides are estimated. After this, part of the sample is centrifugalized and the sediment examined microscopically for casts, red and white blood cells, epithelial cells, crystals of various kinds, etc. If pus is found, smears and cultures are made from samples collected under sterile precautions, and the offending organism isolated. From these cultures of bacteria autogenous vaccines are made in suitable cases. Frequently organisms suspected of being tubercle bacilli are found in samples of urine. In these cases animals are inoculated with the urine and watched for symptoms of tuberculosis.

A careful urine analysis is often the key to the solution of an obscure case. At the present time the laboratory averages three hundred single samples and four hundred twenty-four hour samples per month, with about thirty urine cultures.

Stools are examined for undigested foods; bile pigments, which are often absent in cases of gall stones; occult blood, which freqently gives a clue to ulcers and malignant growths in the intestinal tract; ova of various parasites, such as tapeworm, hookworm, round worms, etc. During the past two years we have found the hook-worm once.

The routine in a blood examination is an estimate of the haemoglobin; counting the number of red and white cells per cubic millimeter; a differential count of the white cells; the color, shape, size, and the presence or absence of parasites in the red cells are noted. In this way diseases with a characteristic blood picture are diagnosed, such as the various forms of anaemias—pernicious, chlorosis, secondary—leukaemias and malaria fever. Some diseases of inflammatory origin, as appendicitis and pneumonia, show an increase in the number of white cells per cubic millimeter, which may be a great help in arriving at a diagnosis; others, of which typhoid fever is an example, show the reverse condition—a diminution in the number of white cells.

Another important examination of blood is the sera diagnosis of syphilis, which is known as Wassermann's reaction. The average for these is about fifty each month.

Cultures of blood are made in various diseases, as septicaemia. These cultures are frequently of great importance, as it is often possible to make a diagnosis of typhoid fever days in advance of the appearance of the Widal reaction.

Cultures are also made from all cases in which pus is found at operation, to ascertain the infecting organism.

Another interesting as well as valuable procedure is the examination of sections of all growths removed at the hospital, to ascertain whether they are malignant (cancer) or non-malignant tumors, or whether they belong to the class known as infectious granulomata. Often in doubtful cases this is done while the surgeon is operating. The surgeon excises a piece of the suspicious tissue, which is immediately frozen by means of carbon dioxid gas, and then very thin sections are cut. These are stained, mounted and examined with the microscope. Frequently it is possible to say in a short time (a few minutes) whether the tumor is cancer or not.

It is hoped that the next few years will bring forth additions to our equipment, further improve our methods, and widen the scope of the laboratory.

### New Committee

THE Trustees have authorized the appointment of certain additional committees to assist in carrying on the work of the hospital. One committee is to have general supervision of the social service work, to raise the funds needed by that department, and to appoint the workers, subject to confirmation by the Board. A second is to have special oversight of the Harrington Hospital. It is charged with the maintenance of the free maternity service, and is to endeavor to build up the private room service. This committee is also to have general supervision of the welfare of all the patients in the Harrington, co-operating with the various agencies created by the social service committee. Though the ground at the Harrington is already covered, in a measure, by the social service workers, the needs of that hospital are special and diverse. The older children there are in themselves a group which a committee could well make its first care.

The chairmen of these committees are to be Trustees, and monthly reports are to be made to the Board.

### The Supply Room

The surgical supplies and dressings for both hospitals are made by the probationary class of the Training School. These pupil nurses receive in the supply room their first lessons in responsibility, as they are in turn placed in charge (under supervision) and held responsible for the making of the supplies and dressings, the care of the room, the economy of material, and the maintenance of a supply sufficient for all regular requisitions and for emergencies. The dressings are sent from the supply room to the operating room for sterilization, and are then ready to be distributed as the requisitions may demand. Neatness and economy in handling materials are taught from the beginning, and are emphasized through the three years of training.

### Gifts

Since the last issue of the BULLETIN, the hospital has received the endowment of a bed in the Harrington Hospital, for a year, from Mr. Franklin D. Locke, one thousand dollars from Mr. Spencer Kellogg, to endow the furnishing of a room, and three thousand dollars on the Stockwell bequest. The bequest from Mr. J. N. Adam has also been announced. These are matters of public congratulation. For until the resources of an institution reach a certain amplitude, it is limited to routine palliative work; whatever strengthens it beyond that point enables it to offer a different quality of service to its community. What a concentration of gifts at a strategic point can accomplish is shown by the maternity service; what returns an instrument of precision can yield let the Pardee Laboratory testify.

AN especially valued gift was announced at the last meeting of the Board of Trustees—the endowment of a bed by Mrs. Charles W. Pardee in memory of her husband.

A TABLE giving the cost per week per patient in thirty hospitals, recently compiled, bears substantial testimony to the good man-vided for and often the families from whom they are temporar-agement of the Buffalo General Hospital. These thirty institutions are of the same general class, but widely distributed geographically. Two are in Canada, the others scattered through thirteen states. The per capita cost at the Buffalo General Hospital is next to the lowest on the list.

---

### Maternity Service.

NEVER has an effort more abundantly justified itself, or have gifts yielded a greater proportionate return than those which established the free maternity service at the Harrington Hospital. It was predicted that this year the service would double, but the increase has been approximately two hundred per cent. Each nurse now attends from twenty to thirty cases, and those in attendance, instead of being "on call" for the twenty-four hours, are now obliged to take the regular day and night shifts. A graduate nurse of experience is always in charge. In consequence of this increased service, nurses are graduating well equipped for obstetric work, and much readier to devote themselves to it than has heretofore been the case. Several young physicians are utilizing the opportunity offered by the Harrington to specialize in obstetrics. Here are substantial results in one department. On the patients' side, two instances may be cited—the first, a woman who had had two children die at birth, but whose third confinement, at the Harrington, was successful; the second, a woman who had lost six children at birth; when, at the Harrington, she was shown her seventh, a living and vigorous child, she could not believe that it was hers. In view of what specialized skill, plus the equipment of a modern hospital, can achieve, it is not surprising that the private maternity service should steadily increase.

The nurses' work with the cases on the free beds is largely educational. These patients are taught the value of baths, general cleanliness and fresh air, and during convalescence they assist in making bandages, folding linen and other such light activities of the ward. Those women who have no homes or means of support are not discharged until they have been provided for, and often the families from whom they are temporarily separated call for the good offices of the social workers. One patient, while under an anaesthetic, carried on an imaginary conversation with her husband. She told him that she would be glad to go to the hospital, where she would be warm and cared for, but how could she leave the other two babies in a cold house, with only one meal a day? It is needless to say that they were promptly cared for.

